# Parental Seasonal Influenza Vaccine Hesitancy and Associated Factors in Shanghai, China, during the COVID-19 Pandemic: A Cross-Sectional Study

**DOI:** 10.3390/vaccines10122109

**Published:** 2022-12-09

**Authors:** Jingyi Fan, Chuchu Ye, Yuanping Wang, Hui Qi, Dong Li, Jun Mao, Hongmei Xu, Xiaojin Shi, Weiping Zhu, Yixin Zhou

**Affiliations:** 1 Department of Epidemiology, School of Public Health, Fudan University, Shanghai 200032, China; 2 Shanghai Pudong New Area Center for Disease Control and Prevention, Shanghai 200136, China; 3 Lujiazui Community Health Service Center, Pudong New Area, Shanghai 200120, China; 4 Zhangjiang Community Health Service Center, Pudong New Area, Shanghai 201210, China; 5 Zhuqiao Community Health Service Center, Pudong New Area, Shanghai 201323, China

**Keywords:** parents, vaccine hesitancy, influenza vaccine, associated factors, COVID-19 pandemic

## Abstract

Background: Seasonal influenza may overlap with the COVID-19 pandemic, and children are one of the priority populations for influenza vaccination in China, yet vaccine coverage has been low. This study aimed to investigate the extent of parental influenza vaccine hesitancy (IVH) and to explore the associated factors. Methods: The study was conducted in Shanghai, China, from 1 June 2022 to 31 July 2022, using an anonymous questionnaire to survey a random sample of parents of children aged six months to 14 years. Binary logistic regression models were used to identify factors associated with IVH. Results: Of the 5016 parents, 34.05% had IVH. Multivariate analysis showed that after adjustment for non-modifiable markers (i.e., sociodemographic, health status, and past vaccination status), being affected by negative influenza vaccine news and having higher “complacency” were positively associated with parental IVH. Higher knowledge of influenza vaccination, being recommended by healthcare workers (HCWs), people around having a positive attitude toward influenza vaccine and having higher levels of “confidence” and “convenience” were negatively associated with parental IVH. Conclusions: In China, public health education aimed at modifying vaccination-related attitudes and beliefs, as well as knowledge and societal influences, could help reduce influenza vaccination hesitancy.

## 1. Introduction

Seasonal influenza is estimated to cause 291,243–645,832 respiratory illness-related deaths worldwide annually [1]. Influenza viruses are prone to change in antigenicity and spread rapidly, with the highest incidence of influenza among children among all age groups and a high disease burden. Children under five years are at higher risk of serious illness after infection [2,3]. Influenza vaccination effectively prevents influenza, reduces influenza-related severe illness and death, and reduces the potential stress on the healthcare system to respond to the COVID-19 pandemic [4]. The Chinese Center for Disease Control and Prevention (CDC) recommends that seasonal influenza vaccination be prioritized annually for key populations, including students in childcare facilities, primary and secondary schools, and children aged six months to five years [5], in line with the position papers of the World Health Organization (WHO) [6] and the U.S. CDC [7].

Despite children being a priority group for immunization, vaccine coverage is poor in China. According to a meta-analysis of 126 studies [8], mainland China’s combined influenza vaccination rate for children aged 6 months to 5 years was 28.4% (95%CI: 23.6–33.2%), less than 30% and generally lower than that of the United States and Japan [9,10,11,12], which failed to create a reliable herd immunity barrier. A previous survey showed that the influenza vaccine coverage rate for children aged 0–14 years in Pudong New Area, Shanghai was about 26.6% in the 2018/2019 influenza season [13].

This low vaccination rate may be due to vaccine hesitancy (VH), which the WHO has included as one of the top 10 global health threats in 2019 [14]. The WHO Strategic Advisory Group of Experts (SAGE) defines VH as the refusal or delay of vaccination when vaccination services are available; it also involves behavioral vaccination but is still a skeptical attitude [15]. It is a worldwide issue that is complicated, context-specific, changes with time, geography, and vaccine type, and has numerous affecting elements [14]. VH has caused a decline in vaccination rates and contributed to the emergence of vaccine-preventable diseases (VPD). In recent years, successive measles outbreaks in some countries in Europe and the U.S. have been closely related to the decrease in vaccination rates caused by VH [16,17]. VH is relatively common globally, with more than 90% of countries reporting some level of VH, and there is low and persistent trust in vaccines regarding vaccination psychology. In recent years, in China, public confidence has declined due to vaccine incidents such as Changchun Changsheng, and there has been a significant increase in parental VH in children after the incident [18,19,20].

One study showed that in the U.S., more parents showed hesitation about the flu vaccine (25.8% hesitation rate) compared to the routine vaccine (6.1% hesitation rate) for children, which is very common [21]. In China, parents were more hesitant about the childhood category B (non-expanded program on immunization (non-EPI)) vaccine than the category A (expanded program on immunization (EPI)) vaccine. A survey in Zhejiang Province, China, showed that 43.2% (95% CI: 40.4–46.0%) of guardians had IVH [22]. A Wuxi, China survey showed that 47.6% of parents hesitated and refused to use the influenza vaccine [23]. Following the COVID-19 pandemic, several studies have indicated that parents are more inclined to vaccinate their children against influenza [24,25,26]. The VH phenomenon may have eased due to the impact of the COVID-19 pandemic. On the other hand, some studies suggest that the COVID-19 pandemic does not appear to be sufficient to encourage children to receive the seasonal influenza vaccine; instead, parents whose children were not vaccinated in the previous year are more hesitant in the future [27].

The “3C” model [15,28] of SAGE integration summarizes the main factors of most VH, including three dimensions of confidence, complacency, and convenience. The “3C” model, as a common factor for VH, should be used in conjunction with specific domestic circumstances, such as uninvolved factors, such as the “accessibility” of the flu vaccine. In addition, the VH determinants matrix provides a comprehensive list of possible factors for VH, including vaccine and immunization program impact, individual and population impact, and situational impact.

IVH can reduce seasonal flu vaccine coverage [29]. In the specific context of the COVID-19 pandemic and childhood COVID-19 vaccination, fewer studies explore multidimensional determinants of IVH among Chinese parents of children under 14. The current IVH status, influenza vaccine knowledge, attitudes, behaviors, and predictors of Chinese parents toward their children during the COVID-19 pandemic are unclear. In summary, the primary study objective of this paper is to explore data on the prevalence and predictors for IVH among parents in Shanghai, China, during the COVID-19 pandemic, which can inform the development of immunization policies to improve influenza vaccine coverage in children.

## 2. Methods

### 2.1. Study Design and Participants

This study was conducted from 1 June 2022 to 31 July 2022 in Pudong New District, Shanghai, eastern China (Figure S1). Participants were fathers or mothers of children aged 6 months–14 years, and only parents were included to ensure that respondents were the decision makers for childhood vaccination. In addition, participants included only permanent residents who had lived in Shanghai for ≥6 months while excluding repeat respondents from the same household.

Our questionnaires were distributed through an online platform (“Questionnaire Star”, www.wjx.cn, accessed on 20 May 2022). We formed a quick response (QR) code for the questionnaire, scanned by all parents on their cell phones via WeChat and self-completed anonymously on their cell phones, with guidance provided by special personnel to answer any possible doubts when completing the questionnaire. A multi-stage cluster random sampling method was used for this study. Based on geographic and population distribution, six sub-centers were divided and established in Pudong New Area, Shanghai.

In the first phase, we randomly selected two communities (legally demarcated) from each of the six sub-centers in Pudong New Area, Shanghai, for a total of 12 sample areas. Meanwhile, the corresponding community health service center (CHSC) in that area, 12 in total, was responsible for the survey within the region.

The second phase had two parts depending on the age of the children: the first part was for parents of children aged 6 months–3 years, who were asked at CHSC immunization clinics when parents brought their children to seek health services, and with informed consent and meeting the inclusion criteria, CHSC staff conducted a face-to-face survey with each parent, and each CHSC was assigned to complete ≥100 questionnaires. In the second part, for parents of children aged 3–14, we randomly selected one kindergarten, one elementary school, and one secondary school in each community, and one class in each grade level, and sampled the cluster as a class. CHSC staff contacted the person in charge of the sampled school and asked them to distribute our questionnaire QR code in the selected class’s parents’ WeChat group and instructed them to fill it in the group.

The Shanghai Pudong New Area Ethics Committee reviewed and approved the study, and all participants completed the questionnaire with informed consent.

For the sample size calculation, a 50% VH rate was expected, the type I error (α) = 0.05, the precision (d) = 0.03, and the design effect (deff) = 2. The minimum target sample size was calculated as 2196 by the formula $N_{min}=deff\times \frac{Z^{2}{}_{\left(1-\alpha \right)/2}\times p\times \left(1-p\right)}{d^{2}}$. Considering a possible 10% non-response rate, a minimum of 2416 individuals would need to be surveyed for this study.

### 2.2. Measures

The questionnaire consisted of four sections.

(1) Basic information on the parents, including age, gender, household location, education level, occupation, income, self-assessed health status, number of children, age of the youngest child, gender of the child, influenza illness history, and influenza and COVID-19 vaccination history.
(2) The extent of IVH was assessed using a five-point Likert question, “Your willingness to get your child vaccinated against influenza in the coming year (2022/2023 influenza season),” which was determined based on responses classified as (i) complete rejecting, (ii) rejecting but still considering (inclined to reject), (iii) undecided or never considered (neutral), (iv) accepted but still considering (inclined to accept), and (v) complete accepted. Respondents judged to have choices 1, 2, 3, and 4 in this study were considered IVH. The question was followed by two additional multiple-choice questions about why the child was not vaccinated last year and about future IVH. In addition, we included the concept of influenza vaccine demand (IVD), meaning complete acceptance of influenza vaccine and actively seeking influenza vaccination services. Those who were utterly receptive and received the influenza vaccine the last year (2021/2022 influenza season) were expected to have “potential” IVD because future vaccination schedules were uncertain and those who had previously received the vaccine showed greater vaccine demand [22].
(3) We used a scale based on the “3C” model (containing the dimensions of confidence, complacency, and convenience) to measure parental IVH characteristics scores [15]. The scale was theoretically guided by the SAGE “3C” model concerning previous studies [22,28] and was revised based on literature reading, resident interviews, and expert consultations. “Confidence” refers to faith in the safety and efficacy of vaccines and the vaccination system. “Complacency” refers to the lack of awareness of the need for and importance of vaccination. “Convenience” refers to vaccine affordability, geographic accessibility, and quality of immunization services. A Likert 5-point scale was used, with complete disagreement, relative disagreement, fair, relative agreement, and complete agreement, corresponding to a score of 1–5, respectively, and items in the complacency section were scored in reverse.
(4) Possible predictors of IVH (such as knowledge, experience to vaccinate, and influence from others and society). For knowledge about influenza infection and the influenza vaccine, we set five questions each, totaling ten. Knowledge about influenza infection in children included concepts, transmission routes, symptoms, and preventive measures; learning about the influenza vaccine, including age, the best time, the interval, and doses of vaccination. The level of knowledge was divided into three levels according to scores: 0 to 1 was low, 2 to 3 was moderate, and 4 to 5 was high. Regarding overall knowledge, a total score of 0 to 2 was low, 3 to 6 was mild, and 7 to 10 was high. In addition, we used the question “Have you heard of the flu vaccine?” to determine “do not know” and a multiple-choice question “What is the main source of information about flu and flu vaccine?” to determine the information channels.

## 3. Quality Control and Statistical Analysis

A completion time <300 s, and continuous multi-point straight-line responses (all options are the same), were treated as invalid questionnaires. The IVH characteristic scale was tested for acceptable reliability and validity, and is detailed in Table S1.

Categorical variables were described using frequencies and percentages. Normally and non-normally distributed continuous variables were analyzed using t-tests and Mann–Whitney U-tests, respectively, to compare the scores of the dimensions of the “3C” factors under different IVH degree groupings, and box plots were drawn. In order to explore the impact of various variables on IVH, binary logistic regression was utilized. The odds ratio (OR) and its 95% confidence interval (CI) were computed on every variable. The Hosmer–Lemeshow test verified the fit of the model. Locally weighted scatterplot smoothing (LOWESS) regression analysis was used to assess trends in the extent of IVH and knowledge of influenza infection and influenza vaccine. Statistical analyses were performed in R (4.2.1) software. In the univariate analysis, variates with a *p* value < 0.25 were incorporated into the multivariate analysis while ensuring the inclusion of gender and age variables. Two-sided *p* < 0.05 was considered statistically significant.

## 4. Results

### 4.1. Sociodemographic Characteristics of the Participants

**Figure 1:**
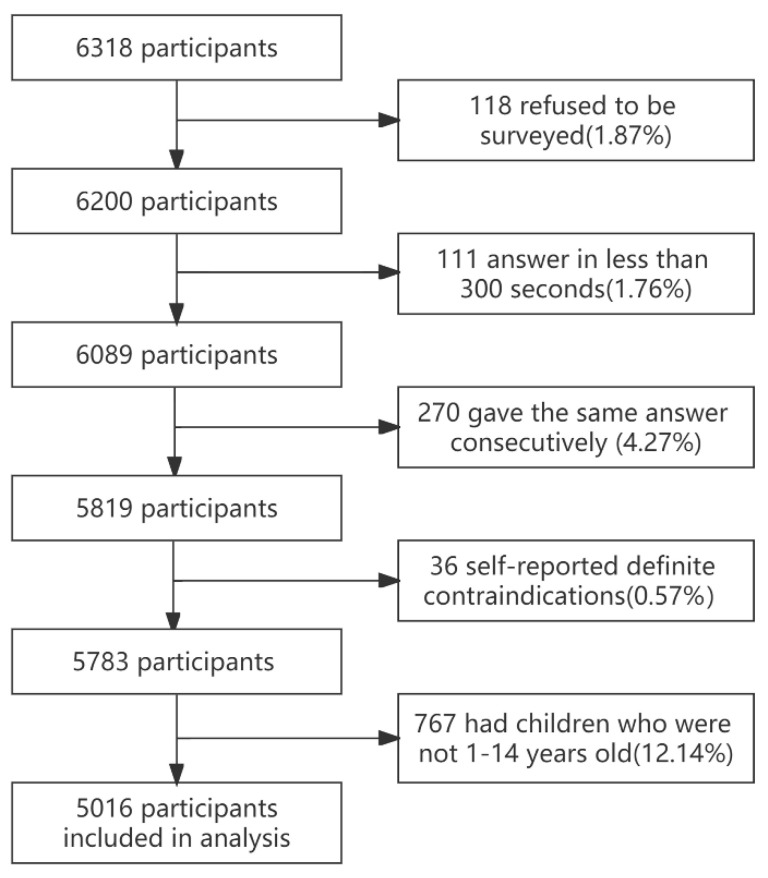
Flow chart of parent questionnaire inclusion.

Our study focused on IVH but given that the analysis included influenza vaccination for the 2021/2022 influenza season, only parents of children aged 1–14 years were included in the data analysis to ensure that children were eligible for immunization (≥6 months of age) in the previous influenza season. Ultimately 5016 valid questionnaires were included in this analysis (Figure 1). The median age of participants was 38 (34–41) years. Here, 76.52% were mothers, 66.85% were Shanghai household registration population, 70.05% had a university degree or higher, 94.00% were not engaged in a healthcare occupation, and the median age of participants’ children was 6 (4–11) years (Table 1).

### 4.2. Parental Influenza Vaccine Hesitancy

**Figure 2:**
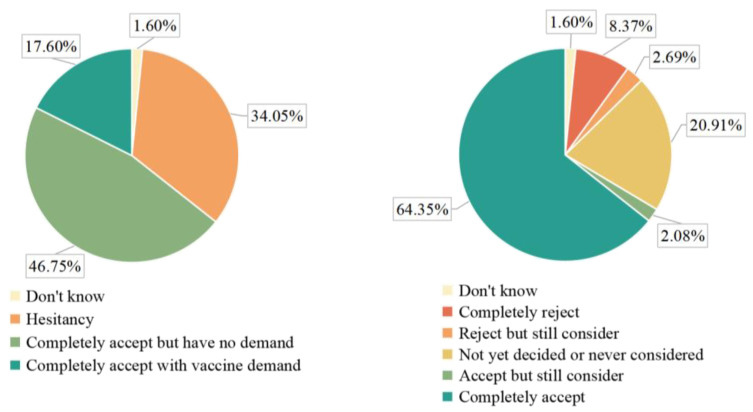
Distribution of parental influenza vaccine hesitation.

Of the 5016 parents included in the analysis, 1708 (34.05%) had IVH. Of these, 420 (8.37%) refused completely, 135 (2.69%) refused but still considered (inclined to refuse), 1049 (20.91%) had not yet decided or had never considered (uncertain and neutral), and accepted but still considering (inclined to accept) (2.08%). The number of parents who fully accepted the influenza vaccine was 3228 (64.35%), while only 883 (27.35%) of the full acceptors had a “potential” IVD (Figure 2).

For the 2021/2022 influenza season, 935 (18.64%) parents reported vaccinating their children against influenza. Eighty (1.60%) parents had never heard of the influenza vaccine.

### 4.3. Predictors of Influenza Vaccine Hesitancy

**Figure 3:**
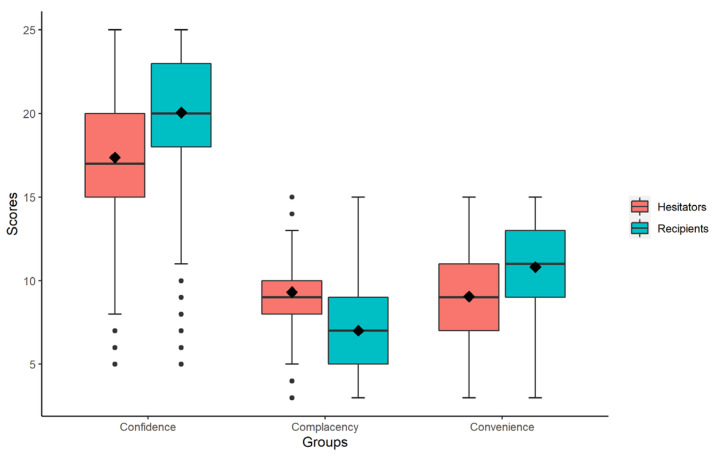
Box plots of dimensional scores of the “3C” model for different IVH subgroups.

A comparison of the scores of the “3C” dimensions between the two groups of parents: IVH and completely vaccine acceptant, based on the presence of IVH, revealed that completely vaccine acceptant scored higher on confidence and convenience and lowered on complacency compared to those with IVH (*p* < 0.001). However, we found no statistically significant difference between the scores on price (*p* = 0.275), and the average score was lower (Table 2 and Figure 3). In addition, we divided the parents into two groups: mild and severe IVH, using the median score of 40 on the “3C” IVH characteristics scale as the cut-off value, and each item of the scale was statistically different between the two groups (*p* < 0.001). The specific scores for each item are shown in Table S4.

Multivariate logistic regression results (Table 3) showed that for sociodemographic characteristics, mothers (OR: 1.506, 95% CI: 1.252–1.813), household registration of other provinces (OR: 0.739, 95% CI: 0.618–0.884), bachelor’s degree or equivalent (OR: 2.126, 95% CI: 1.549–2.920), and master’s degree or above (OR: 2.091, 95% CI: 1.413–3.096) compared to those with lower secondary education or less, children aged 7–12 years (OR: 1.923, 95% CI: 1.419–2.605), 12–14 years (OR: 2.372, 95% CI: 1.671–3.368) compared to the <3 years age group and those who self-rated their child’s health as fair or frail (OR: 2.302, 95% CI: 1.608–3.295) were at higher risk of having IVH.

In terms of past vaccination experience and willingness, parents whose children had received the influenza vaccine in the previous influenza season (OR: 0.129, 95% CI: 0.093–0.179), one dose (OR: 0.589, 95% CI: 0.423–0.819), and two doses (OR: 0.666, 95% CI: 0.544–0.815) of the COVID-19 vaccine had a lower risk of IVH.

**Figure 4:**
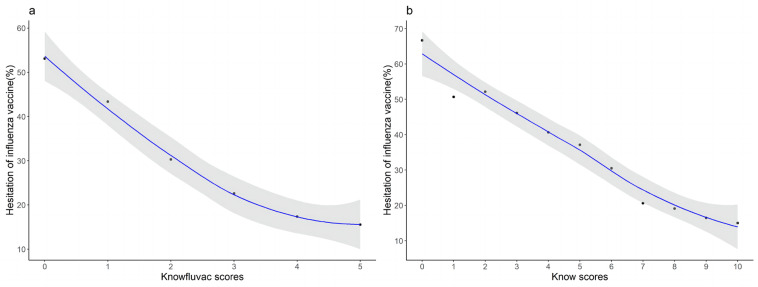
LOESS regression analysis of the trend of knowledge score and IVH proportion: (**a**) knowledge score on seasonal influenza vaccine; (**b**) knowledge score on the total.

Regarding knowledge, parents with moderate (OR: 0.594, 95% CI: 0.499–0.706) or high (OR: 0.567, 95% CI: 0.414–0.776) influenza vaccine knowledge scores were at lower risk of having IVH than parents with low knowledge scores. LOWESS regression analysis showed that the proportion of IVH significantly decreased with increasing knowledge scores (Figure 4).

Influence from others and society: parents who had been influenced by negative news about the influenza vaccine (OR: 1.676, 95% CI: 1.377–2.040) were at higher risk of having IVH. Parents whom a doctor had recommended (OR: 0.812, 95% CI: 0.676–0.976) and had been influenced by people around them to give their children the flu vaccine (OR: 0.630, 95% CI: 0.523–0.758) had a lower risk of having IVH.

Regarding the “3C” factors, moderate or high levels of confidence (moderate: OR: 0.794, 95% CI: 0.657–0.959; high: OR: 0.659, 95% CI: 0.522–0.832), moderate or high levels of convenience (moderate: OR: 0.292, 95% CI: 0.240–0.356; high: OR: 0.309, 95% CI: 0.252–0.380) had a lower risk of having IVH. Moderate or high levels of complacency (moderate: OR: 3.330, 95% CI: 2.605–4.257; high: OR: 7.314, 95% CI: 5.709–9.371) had a higher risk of having IVH.

### 4.4. Information Channels, Reasons for Not Being Vaccinated Last Year, and Future IVH

The results of the multiple-choice questions showed that the primary ways for parents to learn about influenza vaccine were brochures and bulletin boards (50.11%), followed by traditional media such as television and news (49.69%), and family, friends or colleagues (46.05%) (Figure S2).

Among the grounds given by parents for not vaccinating their kids against the flu during the previous flu season, the main one was convenience, with the highest percentage of “transportation inconvenience or lack of time” (29.75%), followed by “fear of adverse reactions and safety after vaccination” (27.53%), and the belief that “it was not worth vaccinating because they would still get the flu after vaccination” (22.83%). On the side, “the impact of COVID-19 vaccination” (5.43%) and the belief that “children are in better health and less likely to get influenza” (4.14%) were also some of the reasons. The percentage of those who did not receive the vaccine because “it was challenging to make an appointment” was 2.25% (Figure S3).

The main reasons given by parents for future IVH were the confidence aspect, “fear of adverse reactions after vaccination” (54.69%), and “fear of vaccine quality and safety” (52.43%). This was followed by the thinking that “it does not prevent all types of influenza” (49.25%), “the child is in better health and less likely to get the flu” (29.90%), and that “the flu is not serious, and the symptoms are mild” (18.02%). In addition, “inconvenient transportation or lack of time” (15.87%), “not knowing how to make an appointment, when and where to get vaccinated” (8.29%), and thinking that “the vaccine is too expensive” (6.66%) were also some of the reasons. The percentage of parents who thought “it was challenging to make an appointment” was 2.32% (Figure S4).

## 5. Discussion

Our study found 34.05% of parents of children with IVH in the 2022/2023 influenza season. Motherhood, local household registration, higher education, older children, self-rated poorer child health, having been affected by negative news about the influenza vaccine and having higher “complacency” were positively associated with parental IVH. On the other hand, children who had received the influenza or the COVID-19 vaccine, had higher knowledge of the influenza vaccine, were recommended by HCWs, had positive attitudes from surrounding people such as family, friends, and colleagues, and had higher “confidence” and “convenience” were negatively associated with parental IVH.

SAGE defines VH as refusing or delaying vaccination when vaccination services are available, and the central issue highlighted is the ambiguity between complete refusal and complete acceptance of vaccination [15]. Our investigation evaluated VH as an attitude using a definition-based item, similar to studies in several countries worldwide [22,30,31]. Some studies have also used VHS scores for assessment [21,32]. By definition, we also included complete rejectors in the IVH. Of the parents with IVH, the largest proportion was neutral, with 20.91% undecided or never considered. Of the 64.35% of parents who fully accepted the influenza vaccine, only 27.35% had a “potential” IVD, having vaccinated their child the previous year. This suggests that while influenza vaccine acceptance is moderate in China, there are still challenges among those who do not seek vaccine services.

Based on the “3C” model theory and concerning previous studies, we designed the IVH trait scale, containing three dimensions: confidence, complacency, and convenience. Our findings showed that the higher the parents’ perceived confidence, including vaccine safety, efficacy, and trust in the vaccine system, the higher the perceived convenience, including ease of transportation and quality of immunization services, and the less hesitant they were to vaccinate their children. In contrast, the lower they perceived the need for vaccination and the severity of the disease, namely, the more complacent they were, the more hesitant they were to vaccinate their children (*p* < 0.001). Furthermore, multivariate analysis showed that these associations persisted even after adjustment for the non-modifiable markers of vaccination hesitancy (i.e., sociodemographic markers, health status, and past vaccination status). The results were generally consistent with previous studies [33], but the difference in perceived price convenience and the item did not differ statistically between the groups with or without IVH (*p* = 0.275). This may be because the influenza vaccine is still a category B vaccine compared to the free vaccine in the EPI program for children, making parents feel that it is overpriced regardless of the presence of IVH and want it to be further reduced or free but perhaps not the main reason for parents’ IVH, which may also be related to the high per capita GDP in Shanghai.

According to the calculated ORs for each item of the scale, the three most important issues were “transportation to the vaccination site is not convenient, or do not have time,” “do not think the vaccine is safe,” and “do not think the flu is serious” attributed to convenience, confidence, and complacency, respectively. In addition, we screened parents of children under five years of age for separate analyses and found the above results to be generally consistent (Table S5). Among the reasons for not vaccinating their children in the 2021/2022 influenza season, the highest percentage was convenience, with 29.75% of parents citing “transportation inconvenience or lack of time,” which may be related to the inconvenience of the closed-off management and concerns about potential infection. The fourth reason for non-vaccination (5.43%) was “received the COVID-19 vaccine”, which may be related to the full launch of the COVID-19 vaccination for children aged 3–11 years in early November 2021 across China. Because simultaneous vaccination with the COVID-19 vaccine and influenza vaccine is currently not allowed (interval > 14 days), the use of regular medical resources for the COVID-19 vaccine may cause inconvenience for influenza vaccination [34]. Future studies could further focus on combined influenza and COVID-19 vaccine regimens for children under 18 years of age to provide more evidence of safety, which is vital for future risk prevention and control of influenza and COVID-19 epidemics [35]. Additionally, according to some research, the influenza vaccine may boost immunity and lessen the severity of COVID-19 [36]. Vaccine safety is one of the most significant predictors of hesitation across the globe [33,37]. We also discovered that the highest proportion of reasons for future IVH among multiple-choice was “worried about adverse reactions after vaccination” (54.69%) and “worried about vaccine quality and safety” (52.43%). It showed that parents’ sentiment toward the safety of the influenza vaccine during the COVID-19 pandemic was still relatively negative, which may have been influenced by the vaccine incident and Internet rhetoric [19].

After model adjustment, we found that mothers are more likely to be flu vaccine hesitant than fathers, consistent with previous studies from multiple sites [23,38,39]. Non-local parents may be more likely to consider the risk of infection for children on their way out and have a lower probability of IVH. Parents with higher education were more likely to have IVH, which differs from studies such as the U.S. [21]. However, a survey of child caregivers in six countries [24] suggests that parents with higher education are less likely to change their minds about vaccination; education may be a proxy marker for other factors influencing adherence to vaccination. In addition, Wuxi, China [23] also showed that parents with less than a university degree were more receptive to the COVID-19 vaccine than parents with a master’s degree or higher education. These suggest the need for effective communication and education targeting mothers of children under 14 years of age, local household registration, and those with a higher degree. We found that parents whose youngest child was of school age (>6 years) were more likely to have IVH, consistent with findings from other regions of China [40]. The study in the U.S. has also shown that influenza vaccine coverage in children in the 5–12 years age group was approximately 33.2% from 2010 to 2017, lower than the 52.6% under-five age group [10]. The result may be because some parents believe that their children’s immunity improves with age, mistakenly believing that the risk of influenza infection and health threat is low, ignoring the need for influenza vaccination or revaccination and creating complacency, thus exacerbating IVH. It may also be related to parents of school-age children no longer paying attention to the category A vaccines (vaccination age generally < 6 years).

The lack of systematic and comprehensive knowledge of influenza infection and vaccines among parents may be one of the reasons behind the phenomenon. Our findings indicated that parents with a higher knowledge score rating of influenza vaccine had a lower risk of having IVH. Another flu vaccine was unknown to 1.59% of parents, thus indicating the need for further propaganda and education to improve parents’ knowledge of the influenza vaccine. Past behavior was an important factor in childhood vaccination. Parents whose children had received the influenza vaccine in the previous season and the COVID-19 vaccine had a lower risk of IVH, which is generally consistent with most countries’ studies [24,37,41], and this may be because parents obtained the protective effect from the vaccine, or it may be related to their own higher health literacy.

Regarding the impact on people and society, parents affected by negative news about the flu vaccine were more likely to have IVH. The media on the Internet may be a double-edged sword, with positive information increasing knowledge and trust and negative and false information doing the opposite. On the other hand, recommendations from HCWs and positive attitudes from colleagues and friends around them could reduce IVH in parents, consistent with previous studies [22]. There are also studies suggesting a significant positive correlation between parents and HCWs regarding vaccine hesitation [23]. These show the future need for HCWs to increase influenza vaccine recommendations based on respect for parental autonomy.

On the side, our study showed that only 18.64% of parents reported vaccinating their children against influenza in the 2021/2022 influenza season, a lower coverage rate than in the U.S. and lower than in local surveys of previous years [13]. Childhood influenza vaccination rates may decline during the COVID-19 pandemic, consistent with studies in the U.S. and elsewhere [42,43]. A study in China has also shown a decline in influenza vaccination rates among Chinese HCWs during the COVID-19 pandemic [34], suggesting a possible general impact of COVID-19. At the same time, there is still the problem of a small number of difficult appointments, which creates IVH. In conclusion, reducing parental IVH and improving influenza vaccine coverage need comprehensive measures.

There are some limitations of this study. First, because it is a cross-sectional survey, only the correlation between the degree of parental IVH and each factor can be determined, not the causal relationship. Second, the respondents of this survey are parents in Shanghai, a relatively high economic level region, and there are certain limitations on the extrapolation of the study results. Third, the questionnaire was self-administered online, and the childhood influenza vaccination status was self-reported rather than based on actual records, which may have been subject to recall bias and overestimated. Finally, the scale has relatively few items in some areas, and more items need to be explored and validated in future studies.

## 6. Conclusions

Our findings suggest that in the Chinese context, public health education aimed at modifying vaccination-related attitudes and beliefs (characterized by the WHO SAGE 3C model), as well as knowledge and societal influences, could help reduce influenza vaccination hesitancy. In the future, further study and disclosure of adverse reactions and protective effects after vaccination, as well as enhanced regulation of vaccine safety, should be made available to enhance parental confidence with more real-world research findings. We need to continue to promote more accurate health education for parents by medical professionals to improve parental knowledge of the flu vaccine and reduce complacency. In addition, the number of flu vaccination sites and daily service hours could be further increased, and simultaneous vaccination could be explored to improve convenience.

## Figures and Tables

**Table 1:** Sociodemographic information of the study subjects (N = 5016).

Sociodemographic Characteristics	N	%
Age (years)		
<30	174	3.69
30–40	2797	59.30
40–50	1658	35.15
≥50	88	1.87
Gender		
Father	1178	23.48
Mother	3838	76.52
Household Registration		
Shanghai	3353	66.85
Other areas (≥6 months of residence)	1663	33.15
Education level		
Junior high school and below	517	10.31
High school or equivalent	985	19.64
Bachelor’s degree or equivalent	2939	58.59
Master’s degree or above	575	11.46
Health care worker		
No	4715	94.00
Yes	301	6.00
Teacher		
No	4815	95.99
Yes	201	4.01
Monthly per capita household income ^2^ (RMB ^1^)		
<2000	124	2.47
2000–5000	572	11.40
5000–10,000	1566	31.22
≥10,000	2754	54.90
Self-assessment of health status		
Healthy or good	4845	96.59
General or weak	171	3.41
Number of children		
1	3160	63.00
2	1719	34.27
≥3	137	2.73
Child’s age (years)		
<3	700	13.96
3–5	828	16.51
5–7	1015	20.24
7–12	1363	27.17
12–14	1110	22.13
Child’s gender		
Male	2680	53.43
Female	2336	46.57
Self-assessment of child health status		
Healthy or good	4772	95.14
General or weak	244	4.86

Note:^1^ USD 1 ≈ RMB 7.0899. The currency-exchange data was retrieved from https://www.chinamoney.com.cn/chinese/index.html (accessed on 14 November 2022, in Shanghai, China). ^2^ The denominator of monthly per capita household income includes all permanent household members, regardless of age.

**Table 2:** Comparison of the scores of each dimension of the “3C” model for different IVH groupings.

Dimensions	Total Score Average	IVH Status		t	*p*	OR (95%CI)
		Vaccine Hesitant (IVH = 1)	Vaccine Acceptant (IVH = 0)			
Confidence	19.13 ± 4.05	17.37 ± 3.76	20.06 ± 3.90	23.329	<0.001	0.839 (0.826–0.853)
Safety	3.64 ± 0.98	3.20 ± 0.92	3.87 ± 0.92	24.179	<0.001	0.470 (0.438–0.503)
Effectiveness	7.58 ± 1.72	6.86 ± 1.62	7.97 ± 1.65	22.657	<0.001	0.672 (0.647–0.698)
Trust in the vaccine delivery system	7.91 ± 1.79	7.31 ± 1.78	8.22 ± 1.72	17.239	<0.001	0.750 (0.725–0.777)
Complacency	7.81 ± 2.56	9.32 ± 2.26	7.01 ± 2.34	−33.649	<0.001	1.549 (1.500–1.599)
No necessity	5.26 ± 1.84	6.36 ± 1.58	4.67 ± 1.69	−34.966	<0.001	1.873 (1.790–1.960)
No severity	2.55 ± 0.97	2.95 ± 0.95	2.34 ± 0.91	−21.807	<0.001	2.017 (1.883–2.161)
Convenience	10.21 ± 2.58	9.06 ± 2.74	10.82 ± 2.26	22.775	<0.001	0.746 (0.726–0.766)
Convenient transportation and time	3.59 ± 1.09	2.92 ± 1.01	3.95 ± 0.96	34.818	<0.001	0.362 (0.338–0.389)
Suitable price	3.02 ± 1.11	2.99 ± 1.18	3.03 ± 1.08	1.092	0.275	0.970 (0.920–1.023)
Good service quality	3.60 ± 1.09	3.15 ± 1.09	3.84 ± 1.02	21.746	<0.001	0.541 (0.510–0.575)

**Table 3:** Logistic regression analysis of predictors of parental IVH.

Characteristics	IVH = 1 n (%)	N	Univariate			Multivariate		
			Unadjusted OR	95%CI	*p*	Adjusted OR	95%CI	*p*
**Sociodemographic characteristics of parents**								
Age (<30 years old)	39 (23.4)	167	Ref			Ref		
30–40	838 (30.5)	2751	1.4	(1.0–2.1)	0.053	0.86	(0.54–1.4)	0.507
40–50	701 (42.9)	1636	2.5	(1.7–3.6)	<0.001	1.1	(0.65–1.7)	0.798
≥50	37 (43.0)	86	2.5	(1.4–4.3)	0.001	1.3	(0.64–2.7)	0.455
Gender (Male)	386 (33.6)	1150	Ref			Ref		
Female	1322 (34.9)	3786	1.1	(0.92–1.2)	0.401	1.5	(1.3–1.8)	<0.001
Household Registration (Shanghai)	1235 (37.4)	3302	Ref			Ref		
Other areas (Residence ≥ 6 months)	473 (29.0)	1634	0.68	(0.60–0.78)	<0.001	0.74	(0.62–0.88)	0.001
Education level (Junior high school and below)	140 (27.8)	504	Ref			Ref		
High school or equivalent	310 (32.0)	969	1.2	(0.97–1.6)	0.096	1.3	(0.97–1.8)	0.073
Bachelor’s degree or equivalent	1035 (35.7)	2896	1.4	(1.2–1.8)	0.001	2.1	(1.5–2.9)	<0.001
Master’s degree or above	223 (39.3)	567	1.7	(1.3–2.2)	<0.001	2.1	(1.4–3.1)	<0.001
Health care worker (No)	1611 (34.7)	4639	Ref			Ref		
Yes	97 (32.7)	297	0.91	(0.71–1.2)	0.468	–	–	-
Teacher (No)	1634 (34.4)	4740	Ref			Ref		
Yes	74 (37.8)	196	1.2	(0.86–1.5)	0.344	–	–	-
Monthly per capita household income (<2000 RNB)	52 (44.1)	118	Ref			Ref		
2000–5000	168 (30.3)	554	0.55	(0.37–0.83)	0.004	0.70	(0.41–1.2)	0.196
5000–10,000	519 (33.6)	1543	0.64	(0.44–0.94)	0.022	0.74	(0.45–1.2)	0.240
≥10,000	969 (35.6)	2721	0.70	(0.48–1.0)	0.062	0.85	(0.51–1.41)	0.526
Number of children (1)	1128 (36.3)	3107	Ref			Ref		
2	538 (31.8)	1694	0.82	(0.72–0.93)	0.002	0.98	(0.82–1.2)	0.785
≥3	42 (31.1)	135	0.79	(0.55–1.1)	0.220	0.93	(0.57–1.5)	0.782
Self-assessed health status (Healthy)	1273 (33.1)	3851	Ref			Ref		
Good	355 (38.7)	917	1.3	(1.1–1.5)	0.001	1.1	(0.87–1.3)	0.581
General or weak	80 (47.6)	168	1.8	(1.4–2.5)	<0.001	1.1	(0.74–1.7)	0.586
Child’s age (<3 years old)	161 (23.5)	686	Ref			Ref		
3–5	208 (25.5)	817	1.1	(0.88–1.4)	0.372	1.3	(0.96–1.8)	0.085
5–7	294 (29.4)	1001	1.4	(1.1–1.7)	0.007	1.3	(0.96–1.7)	0.090
7–12	524 (39.1)	1342	2.1	(1.7–2.6)	<0.001	1.9	(1.4–2.6)	<0.001
12–14	521 (47.8)	1090	3.0	(2.4–3.7)	<0.001	2.4	(1.7–3.4)	<0.001
Child’s gender (Male)	907 (34.3)	2641	Ref			Ref		
Female	801 (34.9)	2295	1.0	(0.91–1.2)	0.681	1.00	(0.86–1.2)	0.990
Self-assessment of the child’s health status (Healthy)	1588 (33.8)	4697	Ref			Ref		
General or weak	120 (50.2)	239	2.0	(1.5–2.6)	<0.001	2.3	(1.6–3.3)	<0.001
**Experience and willingness to get vaccinated**								
Has the child had the flu in the last three years (No)	1389 (34.3)	4055	ref			Ref		
Yes	319 (36.2)	881	1.1	(0.94–1.3)	0.269	–	–	-
Did the child receive a flu vaccine for the 2021/2022 flu season (No)	1656 (41.4)	4001	ref			Ref		
Yes	52 (5.6)	935	0.08	(0.06–0.11)	<0.001	0.13	(0.09–0.18)	<0.001
The number of doses of the child’s COVID-19 vaccine (0 dose)	698 (32.8)	2129	ref			Ref		
1 dose	90 (29.3)	307	0.85	(0.65–1.1)	0.225	0.59	(0.42–0.82)	0.002
2 doses	920 (36.8)	2500	1.2	(1.1–1.3)	0.004	0.67	(0.54–0.82)	<0.001
Have you ever Refused or postponed free vaccinations for your child, except for illnesses or allergies (No)	1109 (31.9)	3479	Ref			Ref		
Yes	316 (37.3)	847	1.3	(1.1–1.5)	0.003	1.2	(0.95–1.4)	0.132
Uncertain	283 (46.4)	610	1.9	(1.6–2.2)	<0.001	1.1	(0.85–1.4)	0.490
Whether they think free vaccines are more important than self-pay vaccines (No)	349 (28.7)	1215	Ref			Ref		
Yes	606 (36.5)	1660	1.4	(1.2–1.7)	<0.001	1.1	(0.90–1.4)	0.315
Uncertain	753 (36.5)	2061	1.4	(1.2–1.7)	<0.001	1.0	(0.78–1.2)	0.679
**Knowledge**								
Score on the flu infection knowledge test (Low)	465 (44.4)	1048	Ref			Ref		
Moderate	791 (36.8)	2147	0.73	(0.63–0.85)	<0.001	0.95	(0.78–1.2)	0.605
High	452 (26.0)	1741	0.44	(0.37–0.52)	<0.001	0.88	(0.70–1.1)	0.252
Score on the flu vaccination knowledge test (Low)	1162 (43.8)	2652	Ref			Ref		
Moderate	462 (26.2)	1763	0.46	(0.40–0.52)	<0.001	0.59	(0.50–0.71)	<0.001
High	84 (16.1)	521	0.25	(0.19–0.32)	<0.001	0.57	(0.41–0.78)	<0.001
**Influences from others and society**								
Have you ever been affected by negative news about the flu vaccine (No)	659 (26.6)	2481	Ref			Ref		
Yes	498 (40.7)	1224	1.9	(1.6–2.2)	<0.001	1.7	(1.4–2.0)	<0.001
Uncertain	551 (44.8)	1231	2.2	(1.9–2.6)	<0.001	1.6	(1.3–1.9)	<0.001
Have you ever received a doctor-recommended influenza vaccine (No)	938 (38.4)	2445	Ref			Ref		
Yes	388 (23.6)	1645	0.50	(0.43–0.57)	<0.001	0.81	(0.68–0.98)	0.027
Uncertain	382 (45.2)	846	1.3	(1.1–1.5)	0.001	1.0	(0.81–1.3)	0.870
Have you ever been influenced by people around you to give your child a flu vaccination (No)	897 (37.9)	2368	Ref			Ref		
Yes	446 (25.0)	1782	0.55	(0.48–0.63)	<0.001	0.63	(0.52–0.76)	<0.001
Uncertain	365 (46.4)	786	1.4	(1.2–1.7)	<0.001	0.90	(0.70–1.2)	0.431
**“3C” Factors**								
Confidence (Low)	857 (50.2)	1581	Ref			Ref		
Moderate	559 (32.7)	1664	0.43	(0.37–0.49)	<0.001	0.79	(0.66–0.96)	0.017
High	292 (17.1)	1691	0.18	(0.15–0.21)	<0.001	0.66	(0.52–0.83)	0.001
Complacency (Low)	134 (7.9)	1449	Ref			Ref		
Moderate	422 (24.7)	1405	4.2	(3.4–5.2)	<0.001	3.3	(2.6–4.3)	<0.001
High	1152 (67.5)	2082	12.2	(10.0–14.8)	<0.001	7.3	(5.7–9.4)	<0.001
Convenience (Low)	624 (36.5)	1041	Ref			Ref		
Moderate	654 (38.3)	1775	0.39	(0.33–0.46)	<0.001	0.29	(0.24–0.36)	<0.001
High	430 (25.2)	2120	0.17	(0.14–0.20)	<0.001	0.31	(0.25–0.38)	<0.001

Note: (i) This analysis did not include parents unaware of the flu vaccine; (ii) the Hosmer–Lemeshow test, chi squared = 10.338, *p* = 0.242.

## Supplementary Materials

The following supporting information can be downloaded at: https://www.mdpi.com/article/10.3390/vaccines10122109/s1, Figure S1: Areas and counties in Shanghai city in China, Table S1: Exploratory factor analysis (EFA) and reliability results of the scale, Table S2: Scores for each item of the IVH characteristics scale (parents of children under 14), Table S3: Comparison of the scores of each dimension of the “3C” model for different IVH groupings (parents of children under 5), Table S4: Logistic regression analysis of predictors of parental IVH (parents of children under 5), Figure S2: Key ways to learn about influenza and the influenza vaccine among parents, Figure S3: Reasons for not getting the influenza vaccine among parents during the 2020/2021 influenza season, Figure S4: Reasons for flu vaccine hesitation among parents for the coming 2021/2022 flu season.
