# Supplementary material for: Parental Seasonal Influenza Vaccine Hesitancy and Associated Factors in Shanghai, China, during the COVID-19 Pandemic: A Cross-Sectional Study
Source: Vaccines (Basel). 2022 Dec 9;10(12):2109. doi: 10.3390/vaccines10122109 (PMC9788477; doi:10.3390/vaccines10122109)
Supplement: Supplementary file 1 [file vaccines-10-02109-s001.zip › vaccines-2063952-supplementary.pdf]

Content

Figure S1. Areas in Pudong New Area in Shanghai.
Table S1. Exploratory factor analysis (EFA) and reliability results of the scale.
Table S2. Scores for each item of the IVH characteristics scale (parents of children under 14).
Table S3. Comparison of the scores of each dimension of the "3C" model for different IVH groupings (parents of children under 5).
Table S4. Logistic regression analysis of predictors of parental IVH (parents of children under 5).
Figure S2. Key ways to learn about flu and the flu vaccine among parents.
Figure S3. Reasons for not getting the flu vaccine among parents during the 2020/2021 flu season.
Figure S4. Reasons for flu vaccine hesitation among parents for the coming flu season.

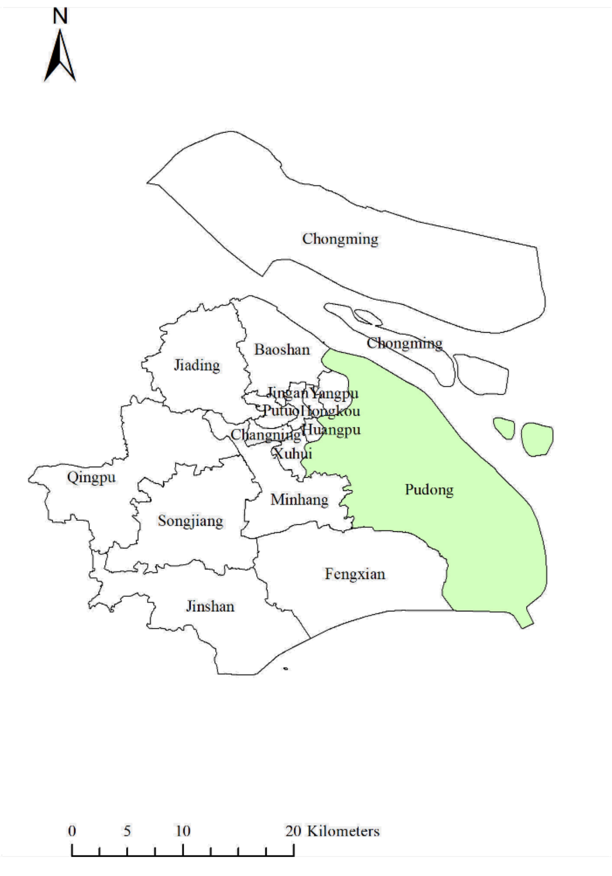

Figure S1. Areas in Pudong New Area in Shanghai.

Table S1. Exploratory factor analysis (EFA) and reliability results of the scale.

Dimensions	Item	Standardized loadings	Communality	Eigenvalues	Variance explained (%)	Cronbach's alpha	CR	AVE
Confidence	L1	0.756	0.637	3.983	36.210	0.909	0.912	0.675
	L2	0.773	0.677					
	L3	0.825	0.726					
	L4	0.883	0.825					
	L5	0.864	0.797					
Complacency	L6	0.614	0.731	2.138	19.436	0.776	0.791	0.563
	L7	0.858	0.752					
	L8	0.758	0.679					
Convenience	L9	0.523	0.594	1.771	16.104	0.682	0.786	0.560
	L10	0.863	0.746					
	L11	0.814	0.729					
Full scale					71.749	0.876		

*Name of each item

L1. I think the flu vaccine is safe.

L2. I believe the flu vaccine can prevent the flu.

L3. I believe the flu vaccine can reduce severe influenza-related illnesses and deaths.

L4. I trust the vaccine-related information provided by the vaccination clinic and the vaccinator.

L5. I believe that healthcare workers.

L6. I think it is necessary to vaccinate children against the flu during the COVID-19 epidemic.

L7. I think my child has a higher probability of getting the flu.

L8. I think the flu is serious.

L9. I have convenient transportation to the vaccination site and have time.

L10. I think the current flu vaccine is overpriced.

L11. I think the environment of the vaccination clinic is poor.

*The overall Cronbach's alpha=0.876 and the Cronbach's alpha for the three dimensions of confidence, complacency, and convenience were 0.909, 0.776, and 0.682, respectively. The KMO value was 0.889, which was greater than 0.8, and Bartlett's test of $P<0.001$. Meanwhile, exploratory factor analysis revealed that the scale consisted of three main factors with a cumulative variance contribution of 71.749%, and the factor loadings of each item are shown in Table S1.

Table S2. Scores for each item of the IVH characteristics scale (parents of children under 14).

Dimension	Items	Total score average	IVH status		t	*P*	IVH characteristic score		t	*P*
			Vaccine hesitant (IVH=1)	Vaccine acceptant (IVH=0)			Mild (≥40)	Severe (<40)		
**Confidence**										
Safety	L1	3.64 ± 0.98	3.20 ± 0.92	3.87 ± 0.92	-24.179	<0.001	4.21 ± 0.75	3.07 ± 0.84	-50.230	<0.001
Effectiveness	L2	3.67 ± 0.95	3.24 ± 0.91	3.90 ± 0.89	-24.296	<0.001	4.24 ± 0.69	3.10 ± 0.82	-52.713	<0.001
	L3	3.91 ± 0.95	3.61 ± 0.93	4.07 ± 0.93	-16.448	<0.001	4.47 ± 0.64	3.35 ± 0.88	-51.013	<0.001
Trust in the vaccine delivery system	L4	3.93 ± 0.93	3.62 ± 0.93	4.10 ± 0.89	-17.406	<0.001	4.52 ± 0.55	3.35 ± 0.86	-57.313	<0.001
	L5	3.97 ± 0.93	3.69 ± 0.93	4.12 ± 0.89	-15.707	<0.001	4.55 ± 0.54	3.39 ± 0.87	-56.524	<0.001
**Complacency**										
Necessity	L6	3.70 ± 1.04	3.03 ± 0.92	4.06 ± 0.92	-37.542	<0.001	4.37 ± 0.72	3.02 ± 0.86	-59.913	<0.001
	L7	3.04 ± 1.07	2.61 ± 0.94	3.27 ± 1.06	-22.561	<0.001	3.49 ± 1.04	2.60 ± 0.89	-31.993	<0.001
Severity	L8	3.45 ± 0.97	3.03 ± 0.92	4.06 ± 0.92	-21.807	<0.001	3.92 ± 0.85	2.97 ± 0.84	-39.720	<0.001
**Convenience**										
Convenient transportation and time	L9	3.59 ± 1.09	2.92 ± 1.01	3.95 ± 0.96	-34.818	<0.001	4.23 ± 0.84	2.95 ± 0.93	-50.641	<0.001
Suitable price	L10	3.02 ± 1.11	2.99 ± 1.18	3.03 ± 1.08	-1.092	0.275	3.32 ± 1.18	2.71 ± 0.95	-20.093	<0.001
Good service quality	L11	3.60 ± 1.09	3.15 ± 1.09	3.84 ± 1.02	-21.746	<0.001	4.14 ± 0.96	3.06 ± 0.94	-39.811	<0.001

*Note: (i) All items in the table are converted to positive wording, i.e., reverse-worded items are reverse-assigned; (ii) See Table S1 for the names of L1-L11.

Table S3. Comparison of the scores of each dimension of the "3C" model for different IVH groupings (parents of children under 5).

Dimensions	Total score average	IVH Status		t	*P*	OR (95%CI)
		Vaccine hesitant (IVH=1)	Vaccine acceptant (IVH=0)			
Confidence	19.70 ± 3.92	17.75 ± 3.74	20.34 ± 3.77	11.464	<0.001	0.846 (0.820-0.874)
Safety	3.74 ± 0.96	3.22 ± 0.97	3.91 ± 0.90	12.007	<0.001	0.470 (0.412-0.537)
Effectiveness	7.80± 1.69	6.96 ± 1.69	8.08 ± 1.59	11.207	<0.001	0.676 (0.628-0.728)
Trust in the vaccine delivery system	8.16 ± 1.72	7.57 ± 1.73	8.35 ± 1.67	7.735	<0.001	0.777 (0.727-0.831)
Complacency	7.30 ± 2.58	9.17 ± 2.43	6.70 ± 2.33	-17.508	<0.001	1.540 (1.540-1.450)
No necessity	4.88 ± 1.85	6.25 ± 1.70	4.44 ± 1.67	-18.035	<0.001	1.866 (1.713-2.033)
No Severity	2.42 ± 0.97	2.92 ± 0.98	2.26 ± 0.92	-11.332	<0.001	2.062 (1.804-2.356)
Convenience	10.64 ± 2.61	9.34 ± 3.05	11.07 ± 2.30	10.001	<0.001	0.769 (0.732-0.808)
Convenient transportation and time	3.78 ± 1.06	2.99 ± 1.07	4.04 ± 0.93	18.033	<0.001	0.370 (0.323-0.423)
Suitable price	3.09 ± 1.14	3.12 ± 1.30	3.08 ± 1.08	-0.508	0.577	1.030 (0.929-1.141)
Good service quality	3.77 ± 1.11	3.22 ± 1.17	3.95 ± 1.02	10.623	<0.001	0.553 (0.495-0.618)

Table S4. Logistic regression analysis of predictors of parental IVH (parents of children under 5).

Characteristics	IVH=1 n(%)	N	Univariate			Multivariate		
			Unadjusted OR	95%CI	*P*	Adjusted OR	95%CI	*P*
**Sociodemographic characteristics of parents**								
Age (<30 years old)	31 (8.4)	143	Ref			Ref		

Characteristics	IVH=1 n(%)	N	Univariate			Multivariate		
			Unadjusted OR	95%CI	*P*	Adjusted OR	95%CI	*P*
30-40	226 (61.3)	1018	1.031	(0.674-1.576)	0.888	1.301	(0.748-2.262)	0.351
40-50	79 (21.4)	218	2.053	(1.265-3.332)	0.004	2.613	(1.346-5.070)	0.005
≥50	2 (0.5)	9	1.032	(0.204-5.221)	0.969	1.162	(0.175-7.702)	0.876
Gender(Male)	89 (24.1)	357	Ref			Ref		
Female	280 (75.9)	1146	0.894	(0.678-1.179)	0.429	1.134	(0.771-1.669)	0.522
Household Registration (Shanghai)	232 (62.9)	917	Ref			Ref		
Other areas (Residence ≥ 6 months)	137 (37.1)	586	0.901	(0.707-1.148)	0.399	-	-	-
Education level (Junior high school and below)	28 (7.60)	103	Ref			Ref		
High school or equivalent	59 (16.0)	249	0.832	(0.493-1.404)	0.490	-	-	-
Bachelor's degree or equivalent	218 (59.1)	923	0.828	(0.523-1.312)	0.422	-	-	-
Master's degree or above	64 (17.3)	228	1.045	(0.621-1.761)	0.868	-	-	-
Health care worker (No)	338 (91.6)	1375	Ref			Ref		
Yes	31 (8.4)	128	0.981	(0.643-1.496)	0.928	-	-	-
Teacher (No)	351 (95.1)	1452	Ref			Ref		
Yes	18 (4.9)	78	0.918	(0.535-1.576)	0.756	-	-	-
Monthly per capita household income (<2000 RNB)	16 (4.3)	44	Ref			Ref		
2000-5000	26 (7.1)	117	0.500	(0.235-1.061)	0.071	0.602	(0.216-1.677)	0.331
5000-10,000	108 (29.3)	443	0.564	(0.294-1.081)	0.085	0.786	(0.319-1.677)	0.602
≥10000	219 (59.4)	899	0.563	(0.299-1.060)	0.075	0.874	(0.360-2.120)	0.766
Number of children (1)	187 (50.7)	795	Ref			Ref		
2	165 (44.7)	652	1.102	(0.866-1.402)	0.431	-	-	-
≥3	17 (4.6)	56	1.417	(0.784-2.564)	0.249	-	-	-
Self-assessed health status (Healthy)	284 (77.0)	1224	Ref			Ref		
Good	71 (19.2)	249	1.320	(0.973-1.792)	0.075	1.362	(0.910-2.038)	0.133
General or weak	14 (3.8)	30	2.896	(1.396-6.006)	0.004	1.541	(0.544-4.367)	0.416
Child's age (1-year-old)	95 (25.7)	402	Ref			Ref		

Characteristics	IVH=1 n(%)	N	Univariate			Multivariate		
			Unadjusted OR	95%CI	*P*	Adjusted OR	95%CI	*P*
2	66 (17.9)	284	0.978	(0.683-1.401)	0.905	1.02	(0.627-1.658)	0.937
3	91 (24.7)	324	1.262	(0.904-1.763)	0.172	1.743	(1.101-2.760)	0.018
4	117 (31.7)	493	1.006	(0.738-1.371)	0.972	1.624	(1.053-2.505)	0.028
Child's gender (Male)	180 (48.8)	778	Ref			Ref		
Female	189 (51.2)	725	1.171	(0.926-1.482)	0.187	1.209	(0.882-1.656)	0.239
Self-assessment of the child's health status (Healthy)	353 (95.7)	1449	Ref			Ref		
General or weak	16 (4.3)	54	1.307	(0.720-2.373)	0.378	-	-	-
**Experience and willingness to get vaccinated**								
Has the child had the flu in the last three years (No)	310 (84.0)	1271	Ref			Ref		
Yes	59 (16.0)	232	1.057	(0.766-1.459)	0.735	-	-	-
Did the child receive a flu vaccine for the 2021/2022 flu season (No)	356 (96.5)	1108	Ref			Ref		
Yes	13 (3.5)	395	0.072	(0.041-0.127)	<0.001	0.083	(0.041-0.166)	<0.001
The number of doses of the child's COVID-19 vaccine (0 dose)	302 (81.8)	1160	Ref			Ref		
1 dose	17 (4.6)	75	0.833	(0.477-1.452)	0.519	0.556	(0.265-1.167)	0.121
2 doses	50 (13.6)	268	0.652	(0.467-0.910)	0.012	0.361	(0.226-0.576)	<0.001
Have you ever refused or postponed free vaccinations for your child, except for illnesses or allergies (No)	240 (65.0)	1114	Ref			Ref		
Yes	74(20.1)	250	1.531	(1.127-2.081)	0.007	1.044	(0.672-1.621)	0.849
Uncertain	55(14.9)	139	2.384	(1.649-3.448)	<0.001	1.398	(0.788-2.480)	0.251
Whether they think free vaccines are more important than self-pay vaccines (No)	80(21.7)	411	Ref			Ref		
Yes	159 (43.1)	534	1.754	(1.291-2.384)	<0.001	1.382	(0.914-2.089)	0.125
Uncertain	130 (35.2)	558	1.257	(0.919-1.719)	0.153	0.715	(0.462-1.105)	0.131
**Knowledge**								
Score on the flu infection knowledge test (Low)	62 (16.80)	141	Ref			Ref		

Characteristics	IVH=1 n(%)	N	Univariate			Multivariate		
			Unadjusted OR	95%CI	*P*	Adjusted OR	95%CI	*P*
Moderate	162 (43.90)	550	0.532	(0.364-0.778)	0.001	0.875	(0.519-1.475)	0.617
High	145 (39.30)	812	0.277	(0.190-0.404)	<0.001	0.747	(0.432-1.292)	0.297
Score on the flu vaccination knowledge test (Low)	219 (59.35)	565	Ref			Ref		
Moderate	125 (33.88)	671	0.362	(0.280-0.468)	<0.001	0.551	(0.385-0.788)	0.001
High	25 (6.78)	267	0.163	(0.105-0.255)	<0.001	0.346	(0.193-0.620)	<0.001
**Influences from others and society**								
Have you ever been affected by negative news about the flu vaccine (No)	143 (38.75)	784	Ref			Ref		
Yes	115 (31.17)	377	1.968	(1.480-2.615)	<0.001	1.330	(0.889-1.990)	0.166
Uncertain	111 (30.08)	342	2.154	(1.612-2.879)	<0.001	1.399	(0.918-2.130)	0.118
Have you ever received a doctor-recommended influenza vaccine (No)	182 (49.32)	639	Ref			Ref		
Yes	120 (32.52)	655	0.563	(0.433-0.732)	<0.001	0.986	(0.685-1.419)	0.940
Uncertain	67 (18.16)	209	1.185	(0.845-1.660)	<0.001	0.861	0.517-1.432)	0.563
Have you ever been influenced by people around you to give your child a flu vaccination (No)	182 (49.32)	643	Ref			Ref		
Yes	108 (29.27)	657	0.498	(0.381-0.652)	<0.001	0.986	(0.685-1.419)	0.003
Uncertain	79 (21.41)	203	1.614	(1.160-2.245)	<0.001	0.861	(0.517-1.432)	0.644
**"3C" Factors**								
Confidence (Low)	208 (56.37)	474	Ref			Ref		
Moderate	85 (23.04)	427	0.318	(0.236-0.429)	<0.001	0.410	(0.274-0.614)	<0.001
High	76 (20.60)	602	0.185	(0.137-0.250)	<0.001	0.716	(0.442-1.159)	0.174
Complacency (Low)	15 (4.07)	352	Ref			Ref		
Moderate	74 (20.05)	469	4.207	(2.371-7.467)	<0.001	4.167	(2.109-8.232)	<0.001
High	280 (75.88)	682	15.642	(9.123-26.819)	<0.001	12.781	(6.467-25.260)	<0.001
Convenience (Low)	134 (36.31)	256	Ref			Ref		
Moderate	133 (36.04)	669	0.226	(0.166-0.308)	<0.001	0.158	(0.103-0.243)	<0.001

Characteristics	IVH=1 n(%)	N	Univariate			Multivariate		
			Unadjusted OR	95%CI	*P*	Adjusted OR	95%CI	*P*
High	102 (27.64)	578	0.195	(0.141-0.270)	<0.001	0.333	(0.213-0.522)	<0.001

*Note: (i) This analysis did not include parents unaware of the flu vaccine; (ii) the Hosmer-Lemeshow test, chi-squared=14.6812, p=0.066.

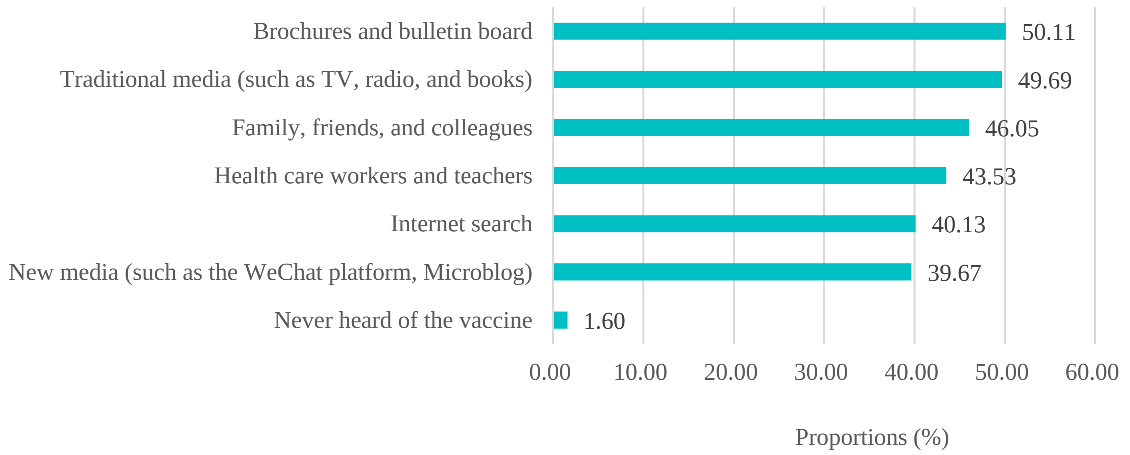

Figure S2. Key ways to learn about flu and the flu vaccine among parents.

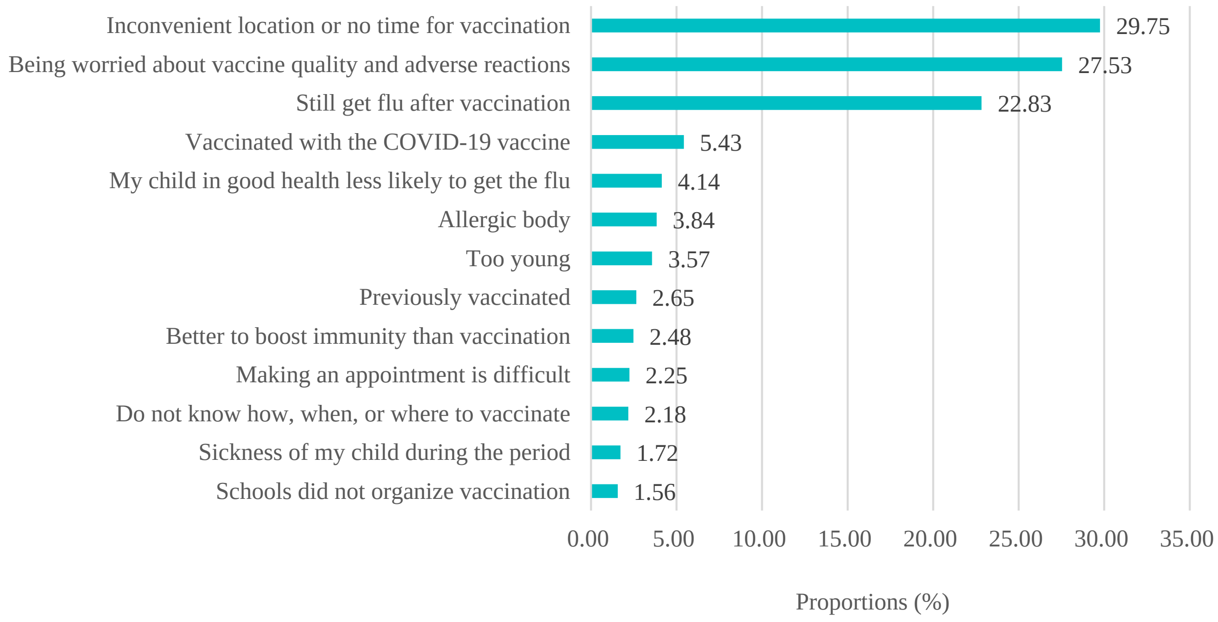

Figure S3. Reasons for not getting the flu vaccine among parents during the 2020/2021 flu season.

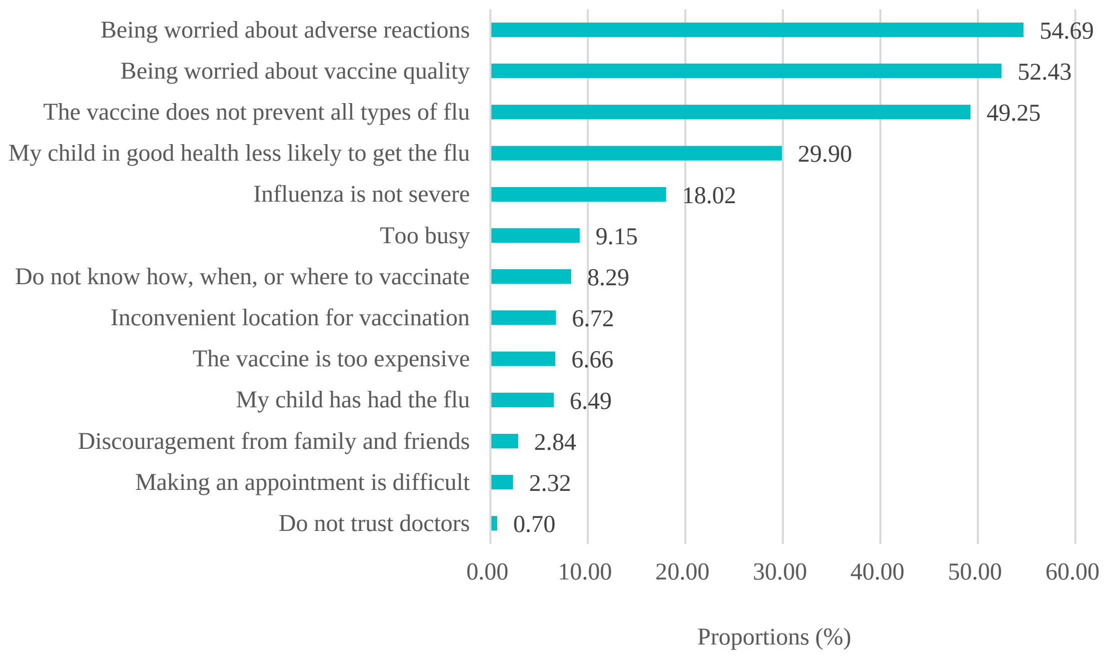

Figure S4. Reasons for flu vaccine hesitation among parents for the coming 2021/2022 flu season.
